# Modulatory Effect of Diosmin and Diosmetin on Metalloproteinase Activity and Inflammatory Mediators in Human Skin Fibroblasts Treated with Lipopolysaccharide

**DOI:** 10.3390/molecules27134264

**Published:** 2022-07-01

**Authors:** Marcin Feldo, Magdalena Wójciak, Aleksandra Ziemlewska, Sławomir Dresler, Ireneusz Sowa

**Affiliations:** 1 Department of Vascular Surgery, Medical University of Lublin, Staszica 11 St., 20-081 Lublin, Poland; 2 Department of Analytical Chemistry, Medical University of Lublin, Chodźki 4a, 20-093 Lublin, Poland; slawomirdresler@umlub.pl (S.D.); i.sowa@umlub.pl (I.S.); 3 Department of Technology of Cosmetic and Pharmaceutical Products, Medical College, University of Information Technology and Management, 35-225 Rzeszow, Poland; aziemlewska@wsiz.edu.pl; 4 Department of Plant Physiology and Biophysics, Institute of Biological Science, Maria Curie-Skłodowska University, Akademicka 19, 20-033 Lublin, Poland

**Keywords:** diosmin, diosmetin, lipopolysaccharide, anti-inflammatory activity, chronic venous disorders, metalloproteinase

## Abstract

Diosmin is widely used as a venoactive drug in the pharmacological treatment of chronic venous disorders. It exerts a strong protective effect on blood vessels via an increase in the elasticity of vessel walls and reduces the permeability of capillary walls, thereby producing an anti-edematous effect. In this paper, we investigated the effectiveness of diosmin and diosmetin in modulating the level of proinflammatory factors in human skin fibroblasts treated with lipopolysaccharide (LPS). Two variants of the experiments were performed: the flavonoid was added 2 h prior to or 24 h after LPS stimulation. Our study revealed that both flavonoids reduced the levels of IL-6 and Il-1β as well as COX-2 and PGE2 but had no impact on IL-10. However, the addition of the compounds prior to the LPS addition was more effective. Moreover, diosmetin modulated the proinflammatory factors more strongly than diosmin. Our investigations also showed that both flavonoids were potent inhibitors of elastase and collagenase activity, and no differences between the glycoside and aglycone forms were observed.

## 1. Introduction

Diosmin and its aglycone diosmetin are polyphenols with a confirmed wide range of biological activities [1,2]; however, the impact of these flavonoids, especially diosmin, on the cardiovascular system has the greatest significance in medicine. In vivo studies have evidenced that diosmin has a strong protective effect on blood vessels via an increase in the elasticity of vessel walls and a reduction in venous pressure and venous stasis. It is also effective in facilitating lymph flow and reduces the permeability of capillary walls, hence its anti-edematous effect [3,4]. Moreover, it alleviates oxidative stress linked with the inflammation process [5]. For these reasons, diosmin is widely used to support the therapy of chronic venous disorders (CVD), and the effectiveness of oral treatment with diosmin has been a subject of numerous studies. CVD includes functional and morphological abnormalities of the venous system manifested by skin lesions, swollen legs, and structural changes in the vein wall, such as varicose veins and venous leg ulcers, which decrease quality of life considerably [4,6].

Dermatological alterations in advanced stages of CVD (C4-C6 according to the CEAP classification), including eczema, discoloration, lipodermatosclerosis, and progressive inflammation, leading to epidermis cracking and cutaneous ulcerations [7], have a significant impact not only on the physical conditions but also on the psychological state of patients, as the associated esthetical concerns often worsen patient’s well-being and may even cause depression [8]. Although pharmacotherapy combined with compression and sometimes surgical intervention is the most effective approach in CVD treatment, topical treatment may alleviate some symptoms and prevent unfavorable processes in skin. Diosmin is commercially available in the form of gel, ointment, or emulsion for topical application; however, scientific reports on its efficacy are scarce [9]. Therefore, the aim of the present paper was to investigate the anti-inflammatory activity of diosmin and its aglycone diosmetin in a human skin fibroblast cell culture treated with bacterial lipopolysaccharide (LPS). Since microenvironmetal changes related to inflammation cause an imbalance in the production of specific pro- and anti-inflammatory cytokines as well as intermediary factors of the arachidonic pathway, which lead to manifestation of skin problems [10,11], the modulatory/regulatory activity of diosmin and diosmetin on the inflammatory mediators, including interleukins 1β, 6,10, cyclooxygenase-2 (COX-2), and prostaglandin E 2 (PGE2), was assessed.

Furthermore, the effect on selected metalloproteinases (MMPs) was studied. MMPs are multidomain zinc-dependent proteinases that degrade various components of the extracellular matrix (ECM) and belong to a superfamily of proteases called metzincins. Typically, MMPs consist of an approx. 80 amino acid propeptide, a catalytic metalloproteinase domain, and an approx. 200 amino acid homopexin domain [12,13]. Type-III collagen is a critical factor for determining the elasticity and distensibility of blood vessels and skin. Some studies have suggested that a decrease in elastin content may cause alterations in vein wall elasticity, leading to CVD with skin lesions [14,15]. Collagenolysis and elastolysis by matrix metalloproteinases (MMP) occur in the tissue remodeling process during inflammatory disease and wound healing. Proteolytic fragments of elastin are highly chemotactic and stimulate inflammation, cell proliferation, and angiogenesis [16]. In this context, chemokines and numerous interleukins (IL-6, IL-1β) may interact, producing MMPs and promoting the inflammation state [17].

## 2. Results

### 2.1. Cell Viability

**Figure 1:**
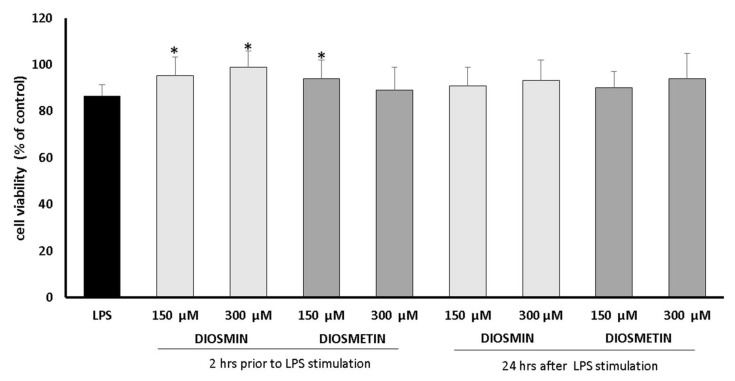
Cell viability determined by the MTT assay and expressed as a % of control (0.5% DMSO). Cells were treated with diosmin and diosmetin 2 h prior to or 24 h after LPS stimulation. The data are means ± SD (*n* = 3). One-way ANOVA followed by Dunnett’s multiple comparison post hoc test; the differences were considered significant at *p* ≤ 0.05. * indicates statistically significant difference.

The initial tests allowed establishing the nontoxic concentration of diosmin and diosmetin for the study. The cellular metabolism (MTT method) and the stability of cell membranes (NR assay) were assessed in a wide concentration range (Supplementary Materials Figure S1). It was shown that diosmin and diosmetin were nontoxic, and even increased cell viability was observed at a diosmin concentration of 150 μM. The subsequent tests aimed to assess the effect of the flavonoids against LPS-induced cytotoxicity. Two variants of the experiments were carried out: diosmin/diosmetin was added 2 h before the LPS stimulation or 24 h after the LPS. Two concentrations of the flavonoids were tested: 150 and 300 μM. The results are shown in Figure 1.

As can be seen, the LPS slightly but in a significant manner reduced the percentage of viable cells to ca. 87%, compared to the control. However, the addition of diosmin/diosmetin 2 h before LPS treatment protected the cells from the negative effect of LPS, and the cell viability was not statistically different compared to the control.

### 2.2. Effect of Diosmin/Diosmetin on Interleukins, COX-2, and PGE-2 in Human Skin Fibroblast Cells Treated with LPS

**Figure 2:**
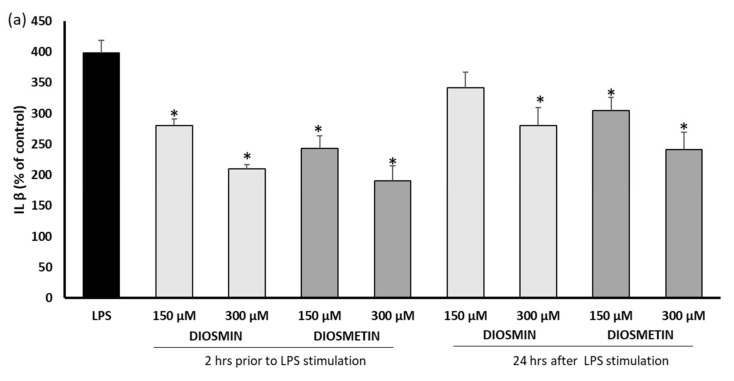

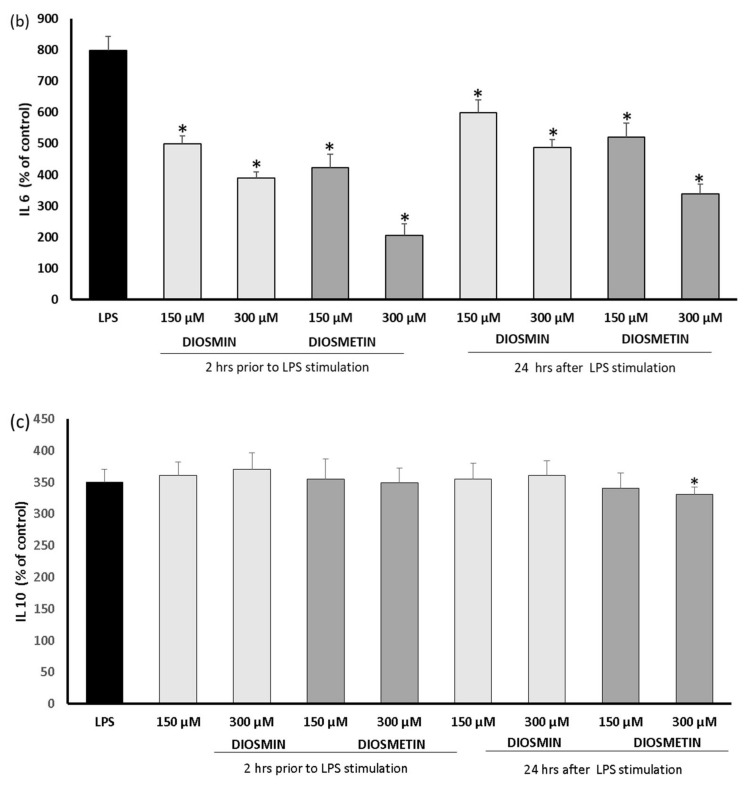
Effect of diosmin or diosmetin on the interleukin level: IL-1β (**a**), IL-6 (**b**) and IL-10 (**c**) in human fibroblast cells treated with LPS. Cells were treated with diosmin or diosmetin 2 h prior to or 24 h after LPS stimulation. The data are means ± SD (*n* = 3). One-way ANOVA followed by Dunnett’s multiple comparison post hoc test; the differences were considered significant at *p* ≤ 0.05. * indicates statistically significant difference.

To assess the anti-inflammatory activity of diosmin and diosmetin, the levels of IL-1β, IL-6, and IL-10 were monitored in fibroblast cells stimulated with LPS (Figure 2). A significant increase (ca 4-fold) in the level of interleukins after the LPS induction was observed, which proved the sensitivity of the cells to LPS. It was found that diosmin and diosmetin significantly reduced the production of proinflammatory interleukins IL-1β and IL-6 in a concentration-dependent manner in both variants of the experiments (Figure 2a,b). However, better results were obtained when the cells were treated with flavonoids prior to the LPS stimulation. Moreover, diosmetin seemed to be more active. This observation confirmed that the sugar moiety in the structure of the compound reduced the anti-inflammatory action. On the other hand, regardless of whether the flavonoids were added prior to or after the treatment of the fibroblasts with LPS, generally no effect on anti-inflammatory IL-10 was observed. The exception was the application of 300 µM diosmetin after the LPS, which slightly decreased IL-10 (Figure 2c).

In the next part of our study, the influence of diosmin/diosmetin on cyclooxygenase 2 (COX-2) and prostaglandin E2 (PGE2) was investigated. Cyclo-oxygenases are intracellular enzymes that catalyze the conversion of arachidonic acid to various forms of prostaglandins (PGs), thromboxanes, and hydroxyeicosatetraenoic acids. Various mitogens may induce inflammation mediated by COX-2 and then changes in COX-2 activity are closely related to PGE2. COX-2 is considered to be linked with many symptoms in CVD, including inflammation, pain, increased angiogenesis, and vascular permeability [18].

**Figure 3:**
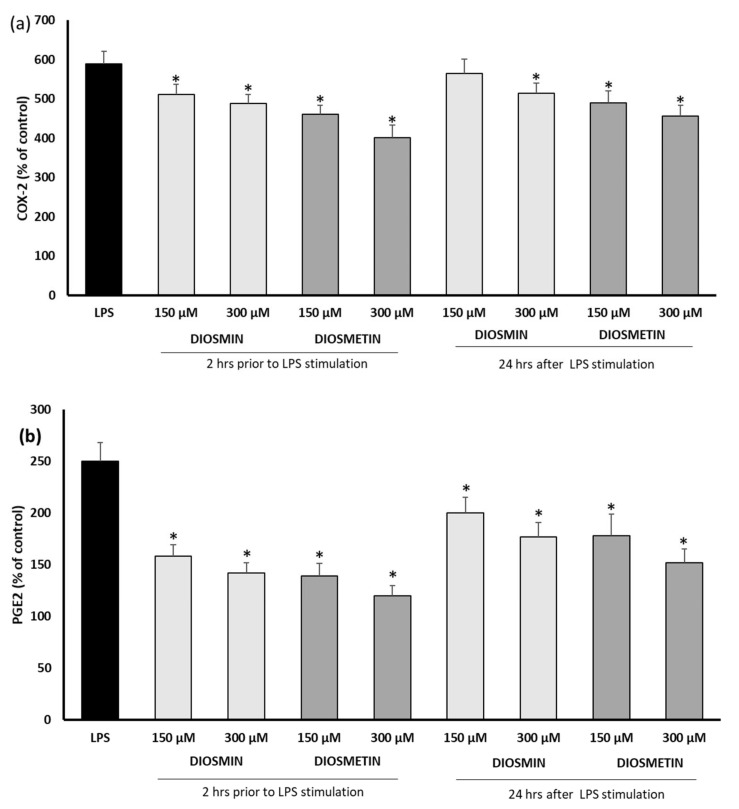
Effect of diosmin or diosmetin on COX-2 (**a**) and PGE-2 (**b**) in human fibroblast cells treated with LPS. Cells were treated with diosmin or diosmetin 2 h prior to or 24 h after LPS stimulation. The data are means ± SD (*n* = 3). One-way ANOVA followed by Dunnett’s multiple comparison post hoc test.; the differences were considered significant at *p* ≤ 0.05. * indicates statistically significant difference.

LPS turned out to be a potent inducer of PGE2 and COX2 (Figure 3) and increased their level ca 2.5-fold and 6-fold, compared to the control. As in the interleukin assay, the flavonoids added before the LPS stimulation acted more effectively; however, it should be noted that the decrease in the COX-2 level was lower than the reduction in PGE-2. This may suggest a different mechanism of modulation of the PGE-2 level.

### 2.3. Effect of Diosmin/Diosmetin on Metalloproteinase Activity

**Figure 4:**
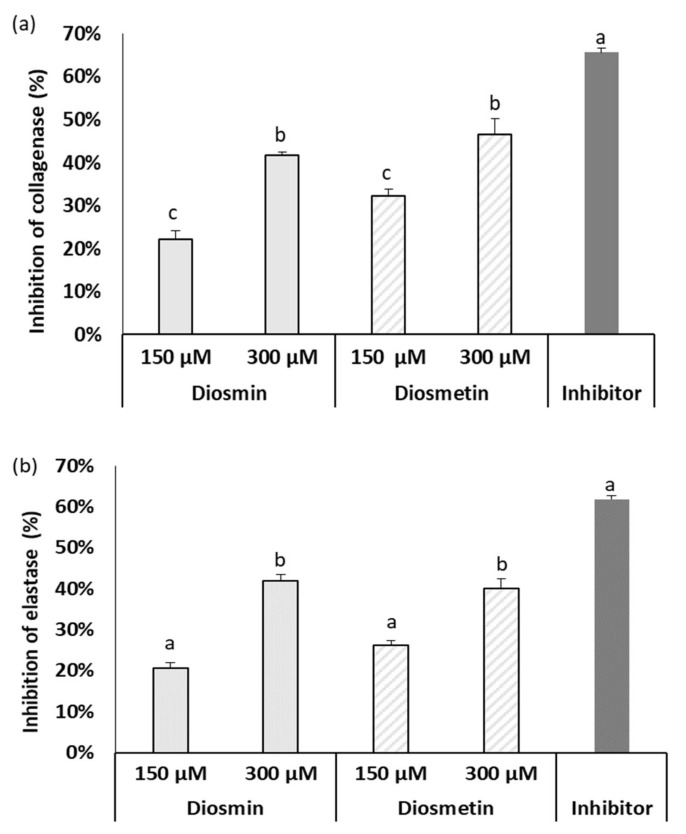
Effect of diosmin or diosmetin on collagenase (**a**) and elastase (**b**) activity. The data are means ± SD (*n* = 3). The lowercase letters, a, b, c, denote significant differences between objects; values followed by the same letter are not significantly different according to Tukey’s test (*p* ≤ 0.05). Succinyl-alanyl-alanyl-prolyl-valine chloromethylketone-SPCK (20 mM) and 1,10-phenanthroline (10 mM) were used as inhibitors of elastase and collagenase, respectively.

Collagen and elastin are key structural proteins in human skin influencing its elasticity and the continuity of the epidermis. The inhibition of matrix metallopeptidases responsible for the degradation of these proteins may protect against unfavorable processes taking place in skin during CVD development. Our investigations revealed (Figure 4) that both flavonoids were strong inhibitors of elastase and collagenase. It is worth noting that the activities of diosmin and diosmetin were similar, which indicates that the aglycone part is responsible for the inhibitory effect on metalloproteinases, and the sugar moiety in the diosmin structure has no impact on metalloproteinase activity.

### 2.4. Antioxidant Assay

**Figure 5:**
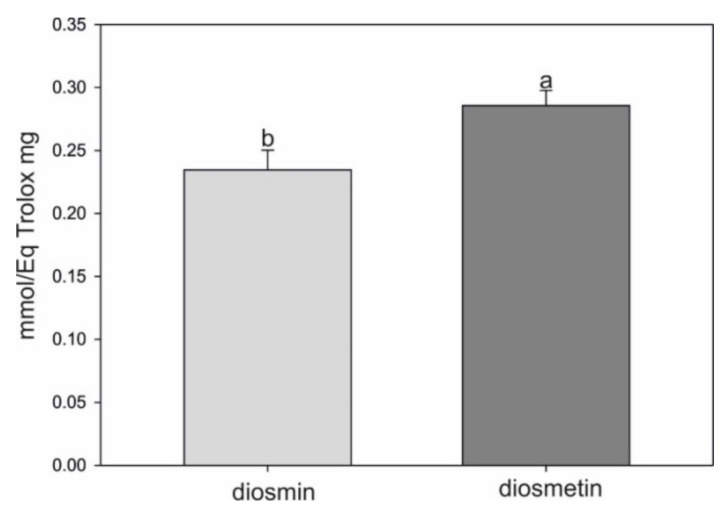
Antioxidant capacity of diosmin and diosmetin measured using the DPPH method and expressed as mM of diosmin or diosmetin per mg of Trolox. The data are means ± SD (*n* = 3). The lowercase a and b denote significant differences between objects according to Student’s test (*p* ≤ 0.05).

Both diosmin and diosmetin were evaluated for their antioxidant capacity using the DPPH assay. The obtained results showed that the aglycone exhibited significantly higher (by approx. 17%) free radical scavenging capacity than the glycoside form (Figure 5). This indicates that the presence of the disaccharide in the structure attenuated the antioxidant activity.

### 2.5. Principle Component Analysis (PCA)

**Figure 6:**
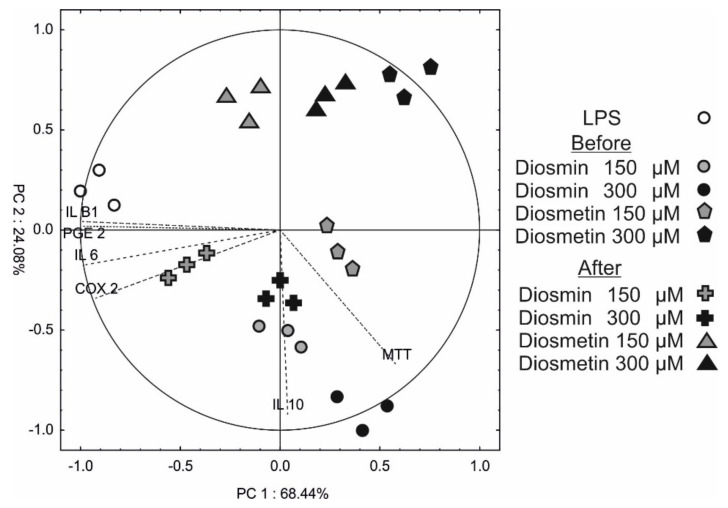
Scatter plot of principal component analysis of MTT, interleukins, PGE-2, and COX-2 in in human fibroblast cells treated with LPS only and with diosmin or diosmetin 2 h prior to or 24 h after LPS stimulation. The length of the dotted lines shows a correlation between original data and PC axes.

To assess the correlation between cell viability, interleukins, PGE-2 and COX-2, and the variant of experiments, principal component analysis (PCA) was conducted. The results are shown in Figure 6.

PC1 and PC2 explained 68% and 24% of total variability, respectively. It was noted that PC1 was negatively correlated with four variables—IL β1, IL 6, COX-2, and PGE-2—while MTT was partially positively correlated with these compounds. The PC1 facilitated the separation of LPS-only individuals from the diosmin and diosmetin treatment samples; however, the samples treated with 150 µM of diosmin or diosmetin 24 h after LPS (located on the left side of the y-axis) were the most similar to the LPS-only samples. IL10 and partially MTT negatively correlated with PC2 grouping all diosmetin-treated samples above the X-axis (except 150 µM before LPS) from the diosmin-treated samples.

## 3. Discussion

Diosmin is widely used as a venoactive drug in the pharmacological treatment of venous disorders. It relieves the symptoms of the disease, improves the quality of the patient’s life, and is well tolerated [18,19,20,21]. In our study, the influence of diosmin/diosmetin on pro- and anti-inflammatory factors in BJ cells treated with LPS was investigated. LPS derived from Gram-negative bacteria was used in our study as one of the most effective inducers of the expression of inflammatory mediators [22]. LPS is a monocyte activator exerting a significant effect on the level of inflammatory factors, including IL-1β, IL-6, TNF-alpha, and IL-8 [23]. It initiates the inflammatory signaling pathway by the activation of the NF-κB factor, and the active form of NF-κB influences the gene transcription of nitric oxide synthase (NOS), COX-2, and cytokines [24].

Our study showed that both flavonoids decreased the level of proinflammatory factors in the in human fibroblast cells treated with LPS; however, the aglycone form (diosmetin) modulated the inflammatory factors more effectively. It significantly decreased the level of proinflammatory IL-B1 and IL-6 as well as COX-2 and PGE2. However, as can be seen, the impact on COX2 was lower than expected. In an in vitro study, Bai et al. suggested that diosmetin stimulated PGE2 via COX-1 more effectively than via COX-2 [25]. A lower effectiveness of diosmin was also observed by Zaragozá et al. [26], where the presence of the disaccharide in the diosmin structure lowered the anti-inflammatory effect. The impact of diosmin on proinflammatory cytokines in an in vivo model was also investigated by Ali et al.; the researchers observed a significant reduction in IL-1β and IL-6 in plasma of diabetic rats [21]. A similar effect was described by Carballo-Villalobos et al. [27].

Some of the features of advanced stages of CVD, i.e., lipodermatosclerosis and/or venous ulcer, may be linked with increased MMP activity and ECM turnover associated with increased mRNA expression and MMP-1 and MMP-2 protein levels [28]. Recent studies have shown an important role of MMPs in the microvascular alteration in chronic venous disease correlated with sub-clinically expressed inflammation, leading to the dilation of veins and skin changes due to the elevated content of MMPs in the vein wall and increased MMP-1 and MMP-9 activity. Changes in the MMP content and activity could lead to tissue remodeling and alterations in the vein wall and adjacent tissue—subcutaneous and dermal layers [29]. Other studies have shown higher counts of monocytes/macrophages, T and B cells, and mast cells further implicated in advanced stages of venous disease [30,31]. These cells engaged in triggering and the development of the inflammation state could be considered as a source of proinflammatory interleukins such as IL-6, IL-10, or IL-1β [32].

Our research revealed a strong modulatory effect of diosmin and diosmetin on elastase and collagenase, as both compounds strongly inhibited these enzymes in a concentration-dependent manner. Interestingly, no differences between the aglycone and glycoside forms were noted.

Collagenase and elastase belong to metalloproteinases (MMP) with proteolytic activity during extracellular matrix (ECM) degradation or remodeling. Collagenases include MMP-1, MMP-2 (neutrophil collagenase), MMP-13, and MMP-18, which are responsible for the specific cleavage of fibrillar collagen types I, II, and III. Elastase degrades elastin and other ECM components and plays a role in tissue remodeling, wound healing, and epithelial cell and macrophage migration [33]. MMPs in skin ulcers cause abnormalities in tissue perfusion and affect angiogenesis and microvasculature, causing disruption in microcirculation in perivascular regions with wound-healing inhibition. The complex interactions between ECM, MMP activity, interleukins, and growth factors in inflammation affected subcutaneous and dermal microarchitecture, leading to progression of CVD stages. Skin damage restoration or wound healing involves an orderly process of re-epithelization matrix deposition and tissue remodeling with an important contribution of MMPs. Skin biopsies from patients with CVD stasis dermatitis (CEAP C4a stage) showed the upregulation of MMPs-1,2 but the downregulation of TIMP-1 in comparison to the control group [33,34]. The MMP-1/TIMP interplay is vital for re-epithelization, while MMP-9 and MMP-13 may be involved in the remodeling of the collagenous matrix [35].

The inhibition of MMP activity by diosmin and diosmetin may protect and stabilize collagen and elastin microarchitecture, which is important in the skin repairing process. Our results showed an inhibitory effect of diosmetin on the release of proinflammatory cytokines and elastolytic activity. The anti-inflammatory action of diosmin was also proved by other researchers. In a study on a DNCB-induced mice model, Lee et al. showed that diosmetin administration induced a decreased infiltration of macrophages in skin changes related to significant reduction in TNF-alpha, IL-4, and Il-1β expression [36]. Diosmetin inhibited the LPS-induced activation of the ERK, p38, and JNK pathways, as well as iNOS expression and NO production [37].

The effect of flavonoids varies depending on their chemical structure and functional group. Diosmetin (aglycone) exerts a relevant cytokine suppressor effect and can enter epithelial cells by passive diffusion because of its increased lipophilicity [38,39]. Our study showed some inhibitory capacities of diosmin on proinflammatory factors and MMP proteolytic activity. However, IL-6 has been shown to stimulate epidermal cell proliferation, and its expression is strongly upregulated in keratinocyte–fibroblast cocultures, in comparison to monocultures. In IL-6-deficient mice, wound healing was severely retarded. In addition, fewer leukocytes and collagen accumulation were observed in these mice [40,41].

These results support the anti-inflammatory effects of diosmin and diosmetin with a noteworthy interaction in proteolytic activity observed in keratinocytes or in fibroblast cell culture. Extracellular matrix (ECM) injury of each constitution also triggers leukocyte infiltration activation and inflammation, which lead to further tissue damage. The molecular determinants that precisely control the dynamics of inflammation complexity during remodeling of ECM, endothelium, vascular wall, and dermal structure, are largely unknown. In this context, understanding of the biochemical and molecular basis of flavonoid interactions on cellular or tissue space and on MMP-induced changes in ECM can provide valuable information on the mechanism of pathogenesis and suggest new treatment strategies. Our study has some limitations, as it was a monoculture experiment. As mentioned above, a coculture cell microenvironment gives a greater possibility to create and note the interplay between keratinocytes and mesenchymal cells. This kind of interactions between keratinocytes, fibroblasts, adipocytes, and microvascular pericytes in the collagen- and elastin-rich extracellular matrix could provide a new insight into the role of polyphenols in anti-inflammatory mechanisms.

## 4. Materials and Methods

### 4.1. Cell Culture

The skin cells used in this study were grown in Dulbecco’s Modification of Eagle’s Medium (DMEM, Biological Industries, Cromwell, CO, USA) with the addition of sodium pyruvate, L-glutamine, 10% fetal bovine serum (Gibco, Waltham, MA, USA), and high glucose content (4.5 g/L). The medium was also enriched with 1% antibiotics (100 U/mL penicillin and 1000 µg/mL streptomycin, Gibco) to prevent microbial contamination. BJs were obtained from the American Type Culture Collection (Manassas, VA, USA). The cells were grown in an incubator in a humidified atmosphere of 95% air and 5% carbon dioxide at 37 °C. When the cells obtained the required confluence, the medium was removed, and the cells were washed twice with sterile PBS (phosphate-buffered saline). After that, the cells were detached from the bottom of the culture flasks with trypsin and placed in fresh DMEM medium. In the next step, the cells were plated in 96-well flat bottom plates and incubated for at least 24 h. After incubation, the cells were treated with diosmin or diosmetin at different concentrations and incubated for 24 h.

### 4.2. Toxicity Assay

#### 4.2.1. MTT Assay

After 24 h, an 3-(4,5-dimethylthiazole-2-yl)-2,5-diphenyltetrazolium bromide solution (5 mg/mL) (Sigma) was added (25 μL/well) and further incubation was conducted for the next 3 h. The crystals were solubilized overnight in a 10% sodium dodecyl sulfate (SDS) in a 0.01 M HCl mixture. Absorbance was measured at 570 nm wavelength using an E-max Microplate Reader (Molecular Devices Corporation, Menlo Park, CA, USA).

#### 4.2.2. Neutral Red Uptake Assay

After 24 h incubation of the cells with the analyzed compounds, the neutral red dye at a concentration of 40 µg/mL was added to the wells. The plates were placed in an incubator for 2 h at 37 °C, the neutral red dye was removed, and the cells were washed with PBS. After this, PBS was removed and 150 µL of decolorizing buffer was added and the absorbance measurements were performed at wavelength λ = 540 nm.

### 4.3. Experimental Design for Analysis of Interleukins, PEG, and Cox-2 in Cells Treated with LPS

The cells were seeded on the well bottom at a density of 1 × 10^5^ cells/mL and incubated for 24 h. To induce the production of inflammatory factors, the cells were stimulated with lipopolysaccharide (LPS) from *Escherichia coli* serotype 0111:B4 (Sigma) (10 μg/mL for 2 h). Diosmin or diosmetin were added to the cell culture 2 h before LPS stimulation and then incubated for 24 h with LPS or 24 h after LPS stimulation and then incubated for another 2 h. After that time, culture supernatants were collected and analyzed. The levels of IL-1β, IL-6, IL-10, COX-2, and PGE2 (Elabscience, Houston, TX, USA) were measured immunoenzymatically (ELISA) using commercially available kits according to the manufacturer’s instruction. The absorbance was measured using a microplate reader at 450 nm. A stock solution of the flavonoid was prepared using DMSO/culture medium (1:1) and it was diluted. The final concentration of DMSO did not exceed 0.5% and this concentration did not affect the cell viability.

### 4.4. Metalloproteinase Activity Assay

#### 4.4.1. Anti-Elastase Activity

To determine the possibility of inhibiting matrix metalloproteinase, a fluorometric neutrophil elastase (NE) kit (Sigma-Aldrich MAK246) was applied. The test was carried out in accordance with the instructions attached to the kit. Succinyl-alanyl-alanyl-prolyl-valine chloromethylketone (SPCK, elastase inhibitor) at a concentration of 20 mM was used as positive control. The analyses were performed in a standard 96-well plate with a clear flat bottom. Fluorescence was measured immediately at excitation wavelength λ = 380 nm and emission λ = 500 nm using a microplate reader (FilterMax F5, Thermo Fisher Scientific, Waltham, MA, USA). The ability to inhibit the NE activity of the analyzed samples was calculated from the following equation:

$$\%\;\mathrm{relative}\;\mathrm{NE}\;\mathrm{inhibition}=\frac{\mathrm{enzyme}\;\mathrm{control}-\mathrm{sample}}{\mathrm{enzyme}\;\mathrm{control}}\;\times \;100\%$$

#### 4.4.2. Anti-Collagenase Activity

To assess the ability of the obtained extracts to inhibit collagenase activity, a fluorometric kit (Sigma-Aldrich, MAK293) was applied. The test was carried out in accordance with the instructions attached to the kit. 1,10-phenanthroline (collagenase inhibitor) at concentration of 10 mM was used as positive control. The analyses were performed in a standard 96-well plate with a clear flat bottom. Absorbance was measured at a wavelength of 345 nm. The measurement was performed in the kinetic mode for 30 min at 37 °C. The ability of obtained extracts to inhibit COL activity was calculated using the following equation:

$$\%\;\mathrm{relative}\;\mathrm{COL}\;\mathrm{inhibition}=\frac{\mathrm{enzyme}\;\mathrm{control}-\mathrm{sample}}{\mathrm{enzyme}\;\mathrm{control}}\;\times \;100\%$$

### 4.5. Antioxidant Activity Assay

The free radical scavenging activity was determined using 2,2-diphenyl-1-picrylhydrazyl (DPPH) according to a method described previously [42]. Trolox equivalent per mM of diosmin or diosmetin was used to express the antioxidant capacity.

### 4.6. Statistical Analysis

The results are presented as means ± SD of three independent measurements (*n* = 3). The data were analyzed using one-way analysis of variance ANOVA followed by Dunnett’s multiple comparison post hoc test to compare the LPS-only group with the treatments. Additionally, Tukey’s post hoc test and Student’s test were performed. Differences were considered significant at *p* < 0.05. All statistical analyses, including PCA, were performed using Statistic ver. 13.3 software (Tibco Software Inc., Palo Alto, CA, USA).

## 5. Conclusions

In this paper, the impact of diosmin and its aglycone, diosmetin, on the level of proinflammatory factors in human skin fibroblast cells treated with LPS and their action on metalloproteinase activity were assessed. Our study showed that diosmin and diosmetin modulated the level of proinflammatory factors and reduced the levels of IL-6 and Il-1β as well as COX-2 and PGE-2 but had no impact on IL-10. Moreover, diosmetin acted more effectively than diosmin, and the addition of the compounds prior to the treatment of cells with LPS was more effective, which suggests the protective activity of these flavonoids on cells. Both flavonoids exhibited moderate free radical scavenging capacity; however, they were strong inhibitors of elastase and collagenase, which may support the skin repairing process during topical application and prevent unfavorable processes in skin associated with CVD development.

## Data Availability

Data is contained within the article or Supplementary Materials.

## Supplementary Materials

The following supporting information can be downloaded at: https://www.mdpi.com/article/10.3390/molecules27134264/s1, Figure S1: Cell viability determined by the MTT assay (a) and the neutral red assay (b). The analyses were performed after 24-h incubation of human fibroblast cells (BJ) with diosmin or diosmetin.
