# Supplementary material for: Modulatory Effect of Diosmin and Diosmetin on Metalloproteinase Activity and Inflammatory Mediators in Human Skin Fibroblasts Treated with Lipopolysaccharide
Source: Molecules. 2022 Jul 1;27(13):4264. doi: 10.3390/molecules27134264 (PMC9268213; doi:10.3390/molecules27134264)
Supplement: Supplementary file 1 [file molecules-27-04264-s001.zip › molecules-1761017-supplementary.pdf]

molecules

Article

Modulatory effect of diosmin and diosmetin on metalloproteinase activity and inflammatory mediators in human skin fibroblasts treated with lipopolysaccharide

Marcin Feldo[1*], Magdalena Wójciak[2*], Aleksandra Ziemlewska[3], Sławomir Dresler[2,4], Ireneusz Sowa[2]

1. Department of Vascular Surgery, Medical University of Lublin, Staszica 11 St., 20-081 Lublin, Poland; martinf@interia.pl
2. Department of Analytical Chemistry, Medical University of Lublin, Chodźki 4a, 20-093 Lublin, Poland; magdalena.wojciak@umlub.pl; slawomirdresler@umlub.pl; i.sowa@umlub.pl
3. Department of Technology of Cosmetic and Pharmaceutical Products, Medical College, University of Information Technology and Management in Rzeszow, Poland, aziemlewska@wsiz.edu.pl
4. Department of Plant Physiology and Biophysics, Institute of Biological Science, Maria Curie-Skłodowska University, Akademicka 19, 20-033 Lublin, Poland.

* Correspondence: martinf@interia.pl Tel.: +48 81 5375944 (M. F.)

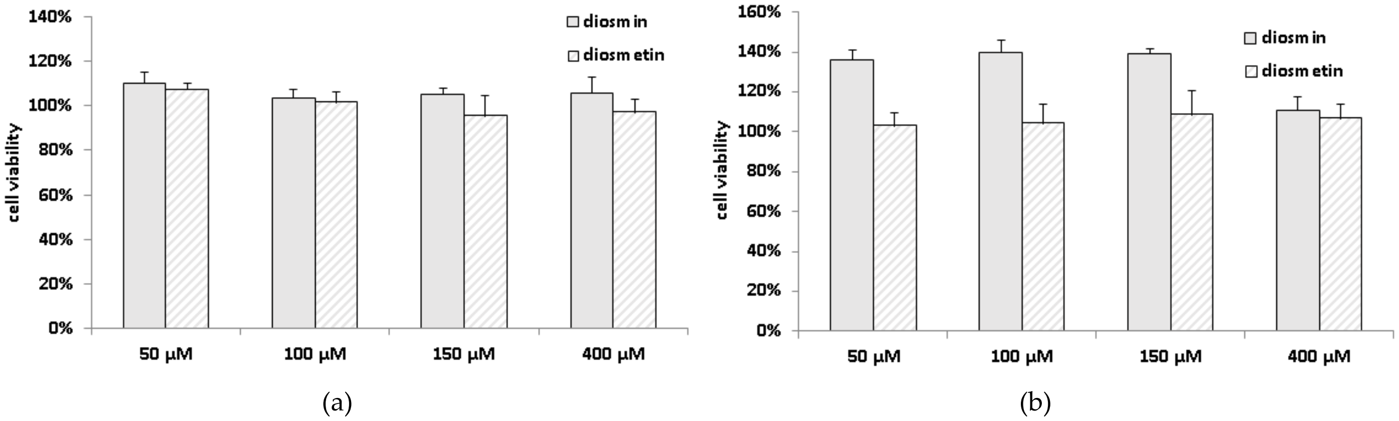

Figure. S1. Cell viability determined by the MTT assay (a) and the neutral red assay (b). The analyses were performed after 24-h incubation of human fibroblast cells (BJ) with diosmin or diosmetin.
